# Impact on mortality of the timing of renal replacement therapy in patients with severe acute kidney injury in septic shock: the IDEAL-ICU study (initiation of dialysis early versus delayed in the intensive care unit): study protocol for a randomized controlled trial

**DOI:** 10.1186/1745-6215-15-270

**Published:** 2014-07-07

**Authors:** Saber Davide Barbar, Christine Binquet, Mehran Monchi, Rémi Bruyère, Jean-Pierre Quenot

**Affiliations:** 1 Service de Réanimation Médicale, CHU de Nîmes – Hôpital Carémeau, Nîmes, France; 2 INSERM, CIE1, CHU Dijon, Centre d’Investigation Clinique –Epidémiologie Clinique (CIC-EC), Dijon, France; 3 Service de Réanimation Polyvalente, CH de Melun, Melun, France; 4 Service de Réanimation Médicale, CHU Le Bocage, & INSERM UMR866, Faculty of Medicine, Dijon, France

**Keywords:** severe acute kidney injury, septic shock, renal replacement therapy, intensive care

## Abstract

**Background:**

One of the most dreaded complications of septic shock is acute kidney injury. It occurs in around 50% of patients, with a mortality rate of about 60% at 3 months. There is no consensus on the optimal time to initiate renal replacement therapy. Retrospective and observational studies suggest that early implementation of renal replacement therapy could improve the prognosis for these patients.

**Methods/design:**

This protocol summarizes the rationale and design of a randomized, controlled, multicenter trial investigating the effect of early versus delayed renal replacement therapy in patients with severe acute kidney injury in early septic shock. In total, 864 critically ill adults with septic shock and evidence of acute kidney injury, defined as the failure stage of the RIFLE classification, will be enrolled. The primary outcome is mortality at 90 days. Secondary outcomes include safety, number of days free of mechanical ventilation, number of days free of renal replacement therapy, intensive care length of stay, in-hospital length of stay, quality of life as evaluated by the EQ-5D and renal replacement therapy dependence at hospital discharge. The primary analysis will be intention to treat. Recruitment started in March 2012 and will be completed by March 2015.

**Discussion:**

This protocol for a randomized controlled study investigating the impact of the timing of renal replacement therapy initiation should provide an answer to a key question for the management of patients with acute kidney injury in the context of septic shock, for whom the mortality rate remains close to 60% despite improved understanding of physiopathology and recent therapeutic advances.

**Trial registration:**

ClinicalTrials.gov identifier NCT01682590, registered on 10 September 2012.

## Background

The incidence of acute kidney injury (AKI) in patients with septic shock is around 50% [1-3]. In severe septic shock, the need for renal replacement therapy (RRT) contributes to increasing the risk of death from less than 40% in septic shock without AKI, to over 60% in the forms associated with severe AKI requiring RRT [4]. It is now proven that AKI associated with sepsis is an independent risk factor for mortality [5,6], not just an epiphenomenon related to the severity of the patients’ illness. The discrimination of septic and non-septic AKI may have clinical relevance for clinicians [7]. Some experimental studies suggested that septic AKI may be characterized by a distinct pathophysiology [8]. Thus, septic AKI may have differences in clinical outcomes and responses to interventions compared to non-septic AKI.

### Initiation of renal replacement therapy: state of the art

In chronic renal failure patients, there is no benefit to initiating dialysis until the glomerular filtration rate drops to less than 7 mL/min (except in case of uremic syndrome). This is usually the beginning of dependence on dialysis for these patients [9]. The situation is different for patients with AKI, and the optimal time to start RRT remains unknown. In current practice, the initiation of RRT is sometimes very early (i.e. within the first 24 hr) [10], but may sometimes be delayed, whereby diuresis is stimulated with diuretics, and RRT is started only with the appearance of serious events, rather than following a proactive decision based on the severity of renal impairment [11]. Indeed, this attitude is justified by the possibility that appropriate early treatment of sepsis may limit the progression to renal failure [12].

In one historical study published in 1960, Teschan *et al*. [13] introduced the concept of prophylactic hemodialysis, advancing the hypothesis that the prevention of uremic syndrome could prevent its complications, including sepsis. In this study, the threshold for defining RRT was a urea level above 57 mmol/L, and dialysis was initiated 2 to 3 days after the diagnosis of AKI. Since this initial study, several authors have compared early versus late therapy, using different criteria and thresholds.

To the best of our knowledge, there is only one randomized controlled trial to date that has examined the benefits of early initiation of RRT in critically ill patients with AKI [14]. In this study, which compared two doses of dialysis, a secondary analysis compared early (within 12 hr of diagnosis) versus late hemofiltration (when the patient fulfilled the conventional criteria for RRT, namely a plasma urea level of >40 mmol/L, potassium of >6.5 mmol/L or severe pulmonary edema). On average, hemofiltration was started 7 hr after inclusion in the early group and a mean of 42 hr after inclusion in the late group. No significant differences were found, either in terms of mortality at 28 days, or in terms of recovery of renal function. However, the study was undersized to answer the question of the timing of RRT (analysis of 106 patients) and was not specifically designed to test this hypothesis.

Several observational and retrospective studies that have been conducted with patients with AKI, with or without sepsis, suggest on the contrary that there may be a benefit to early initiation of RRT [15-17]. A meta-analysis published in 2008 by Seabra [18], which included studies published over the last five decades, found a statistically significant reduction in mortality with early RRT in 18 cohort studies, with a relative risk of 0.72 (95% confidence interval 0.64 to 0.82, *P* < 0.001). In one prospective, multicenter, observational study [19], the timing of RRT was stratified into early and late by median urea (24.2 mmol/L) and by median creatinine (309 μmol/L) at the time RRT was started. There was no significant difference in adjusted hospital mortality when stratified by median serum urea at the time of RRT initiation. Conversely, when stratified by creatinine levels, late RRT was associated with lower mortality. Timing was also categorized temporally from ICU admission into early (<2 days), delayed (2 to 5 days) and late (>5 days), and late RRT was associated with increased mortality.

Other authors have tried to initiate very early RRT as a treatment for the initial phase of septic shock, with discordant results [20,21]. In these studies, hemofiltration techniques were introduced before the onset of established AKI, more with a view to immunomodulation than to treating the AKI itself. Interestingly, a recent retrospective multicenter observational study [22] found a U-shaped association between the timing of RRT initiation and in-hospital mortality, with reduced mortality when initiation of RRT was between 2 and 3 days after ICU admission. The peak mortality when RRT was initiated very early was related to the extreme severity of disease in these patients, whereas age and septic complications were responsible for the highest mortality in the case of late RRT (after D4).

The overall results of these studies would seem to indicate that there is a benefit from early initiation of RRT, but the exact definition of early remains to be determined, as does the definition of the criteria for AKI necessitating RRT. The question of the right time to begin RRT remains unanswered and controversial, and poses a real problem in the management of patients with septic shock and AKI. In this context, we propose a randomized, multicenter, controlled trial on the impact on mortality of the timing of RRT initiation in patients with acute kidney failure in septic shock.

### Objectives

The primary objective is to assess whether the timing of RRT initiation (early versus delayed) has an impact on mortality at 90 days in patients with AKI at the failure stage according to the RIFLE criteria [23] (see details below in Study definitions), during the initial phase of septic shock. The secondary objectives are: (1) to evaluate the impact of early versus delayed initiation of RRT on key secondary endpoints including: 28-day, 180-day and 1-year mortality; number of days free of mechanical ventilation; number of days free of RRT; ICU length of stay; hospital length of stay; and 90-day and 1-year quality of life as evaluated by the EQ-5D questionnaire; (2) to compare the efficacy and safety of the two strategies in terms of episodes of metabolic disorders, arrhythmia, pulmonary edema, hypotension, hemorrhage and RRT dependence at hospital discharge.

## Methods/design

### Study design, setting and patient population

This is a randomized, controlled, open-label, multicenter study comparing two strategies for the management of AKI occurring in patients in the initial phase of septic shock: namely initiation of RRT within 12 hr of the onset of AKI versus initiation of RRT after 48 to 60 hr in the failure stage of the RIFLE classification [23] (see details below in Study definitions), or at the onset of criteria requiring emergency RRT. The study will be conducted in 24 ICUs (15 university teaching hospitals and 9 general (non-academic) hospitals) in France. All patients admitted to the ICUs of participating centers will be screened for eligibility. The study sponsor is the University Hospital of Dijon, France, where data will be managed by the Centre for Clinical Investigation and Clinical Epidemiology (CIC-EC).

### Inclusion criteria

• Age >18 years.

• Patients in the first 48 hr of septic shock developing AKI with at least one criterion characteristic of the failure stage of the RIFLE classification (defined below under Study definitions).

• Informed consent provided by the patient (or person with decisional responsibility).

• Patients must have social security cover.

### Exclusion criteria

The following conditions will lead to ineligibility for study entry:

• Chronic RRT.

• Obstructive etiology for AKI.

• Need for emergency RRT before randomization (hyperkalemia >6.5 mmol/L, metabolic acidosis with pH < 7.15 or extravascular fluid overload refractory to diuretics with pulmonary edema).

• AKI that has already been treated by RRT in the ICU.

• Confirmed or suspected pregnancy (verified by serum [b-HCG] pregnancy test if necessary).

• Patient is moribund with expected death within 24 hr.

• Patients for whom survival to 28 days is unlikely due to an uncontrollable comorbidity (cardiac, pulmonary or hepatic end-stage disease; hepatorenal syndrome; poorly controlled cancer; severe post-anoxic encephalopathy; etc.).

• Patients with advance directives issued expressing the desire not to be resuscitated.

• Patient under tutorship, curatorship or judicial protection.

• Enrollment in any concomitant randomized trial with mortality as a primary outcome.

### Study definitions

#### Septic shock

Septic shock is defined according to current guidelines [24,25] as sepsis-induced hypotension persisting despite adequate fluid replacement, requiring the initiation of vasopressor therapy. The initial phase of septic shock is defined as the first 48 hr after the start of vasopressor therapy.

#### Sepsis

The operational definition of the clinical syndrome of sepsis will be confirmed or suspected infection, and at least two systemic inflammatory response syndrome criteria, i.e. any two of the following: temperature >38°C or <36°C; heart rate >90 beats/min; respiratory rate >20 breaths/min, PaCO_2_ < 32 mmHg or mechanically ventilated; white cell count >12,000 cells/mm^3^, <4,000 cells/mm^3^ or with >10% immature (band) forms.

#### Acute renal failure

AKI will be defined and classified according to the RIFLE criteria (risk of renal dysfunction, injury to the kidney, failure of kidney function, loss of kidney function and end-stage kidney disease) as outlined by the Acute Dialysis Quality Initiative (ADQI) Working Group [23]. In brief, the RIFLE criteria classify AKI into three categories of severity (risk, injury and failure) and two categories of clinical outcome (loss and end-stage kidney disease) based on relative changes to serum creatinine and urine output. The presence of AKI will be defined by the failure stage of the RIFLE classification, namely an abrupt (within 7 days) reduction in kidney function, characterized by a threefold increase in serum creatinine relative to baseline; an absolute value ≥354 μmol/L (accompanied by an acute increase ≥44.2 μmol/L); a reduction in urine output of ≤0.3 mL/kg/hr for ≥24 hr; or anuria for ≥12 hr. If pre-hospital baseline serum creatinine is unavailable, baseline serum creatinine is estimated based on back-calculation of the abbreviated Modification of Diet in Renal Disease (MDRD) as recommended by the ADQI [23].

### Treatment

#### Renal replacement therapy

RRT will include any form of extracorporeal RRT for patients with documented AKI. Given the lack of demonstrated superiority of continuous over intermittent techniques, and of convective over diffusive techniques [26,27], investigators at each center will be free to choose the extra-renal purification technique based on their usual practice (intermittent hemodialysis, intermittent sustained low-efficiency dialysis (SLED), continuous hemodialysis, continuous hemofiltration or continuous hemodiafiltration) and can move from one technique to another depending on the needs of the patient (typically the continuous technique in the acute phase, followed by intermittent techniques after stabilization).

Vascular access is obtained through a double lumen catheter with a minimum diameter of 12 French, preferably implanted in the superior vena cava via the jugular approach, and whose end is positioned 1 cm from the junction between the superior vena cava and the right atrium, or alternatively at the inferior vena cava by the femoral route. Catheter placement under ultrasound guidance is strongly recommended but not mandatory. Investigators must follow international guidelines on the management of AKI [28,29] to standardize practices and optimize metabolic control and hemodynamic stability during treatment.

For continuous techniques, investigators must provide treatment continuously for 24 hr with a change of membranes at least every 72 hr. To be certain of administering the recommended dialysis dose despite interruptions for changing filters or for additional procedures away from the ICU, investigators must regulate a minimum ultrafiltration (or dialysate) rate of 25 mL/kg/hr.

For intermittent techniques, the recommendations are to set a blood flow rate of 150 to 250 mL/min, a dialysate flow rate of 300 to 500 mL/min, a high concentration of sodium in the dialysate (150 mmol/L) and a low temperature of the dialysate (35°C). Treatment should be initiated with an isovolemic connection (simultaneous connection of two lines filled with saline). The length of sessions must be at least 4 hr and preferably 6 hr or more. The frequency of sessions should be at least once every 48 hr, or more often if investigators deem it necessary to achieve optimal metabolic control, a urea concentration <30 mmol/L or a stable salt and water balance. Investigators will be required to use biocompatible membranes.

Investigators can use systemic anticoagulation with unfractionated heparin or low molecular weight heparin, or alternatively, regional citrate anticoagulation.

#### Early renal replacement therapy

The operational definition of early RRT is the initiation of RRT immediately after the diagnosis of AKI. A maximum of 12 hr is allowed between the diagnosis of AKI and the actual initiation of RRT, given the time required for dialysis catheter placement and installation of equipment for RRT, and allowing for technical difficulties that may be encountered in clinical practice.

#### Delayed renal replacement therapy

The operational definition of delayed RRT is the initiation of RRT at least 48 hr after the diagnosis of AKI. A maximum margin of 12 hr (i.e. up to 60 hr) is allowed before actual initiation of RRT, justified by the same technical reasons mentioned above for the early RRT group.

#### Emergency renal replacement therapy

By protocol and to minimize the potential bias of clinician discretion on when to initiate emergency RRT, at least one of the following criteria must be fulfilled prior to initiation of emergency RRT:

1) hyperkalemia ([K^+^] ≥6.5 mmol/L) with characteristic electrocardiographic changes

2) metabolic acidosis (pH <7.15) defined as a base deficit > 5 mEq/L or HCO_3_^–^ < 18 mEq/L

3) pulmonary edema

#### Renal recovery

Renal recovery is defined as the return of spontaneous urine output ≥1,000 mL/24 hr (or ≥2,000 mL/24 hr with diuretics) for a minimum of 24 hr without RRT.

### Trial protocol

#### Description of study flow

**Figure 1:**
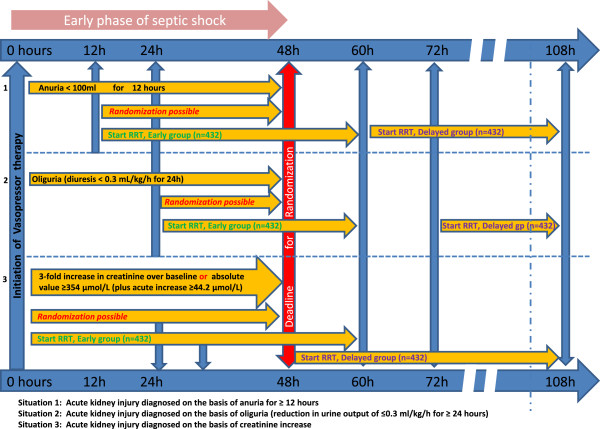
**Flow chart of the study groups.** The timing of diagnosis, randomization and initiation of renal replacement therapy in the early and delayed groups, according to the method used to diagnosis acute kidney injury, are shown.

Patients will be identified in the ICU through daily surveillance by the research coordinator or treating ICU physician. Each patient’s eligibility will be verified by use of a checklist that summarizes the inclusion and exclusion criteria (Figure 1). Septic shock is considered as confirmed once vasopressor therapy is initiated. For patients diagnosed with septic shock, AKI must appear within 48 hr for the patient to be eligible (in addition to the failure criteria).

#### Sample size calculation

It has been shown that the mortality of septic shock patients who develop AKI is around 50%. Assuming an absolute reduction in mortality of 10% (55% mortality at 90 days in the delayed RRT group versus 45% in the early RRT group), 864 patients (432 per group) are required to ensure 80% power at a bilateral alpha risk of 0.05, assuming a rate of 5% non-evaluable cases.

The expected reduction of 10% in mortality was chosen as a clinically relevant difference by the study team. There are currently no data in the literature that could serve as a precedent for this, due to wide variations between existing studies in the types of patients included, the definitions of AKI used, and the definition of what constitutes early or late treatment. In light of the heterogeneous data available, a conservative estimate of a 10% reduction was retained as being clinically relevant.

#### Informed consent

When patients meet the inclusion criteria, and do not present any non-inclusion criteria, they are informed (orally, with supporting documentation in written format) about the study by the investigators, and invited to participate. If the patient is temporarily incapable of receiving the appropriate information or making an informed decision regarding consent to participate, they can still be included if informed consent is given by the patient’s surrogate or legal representative. If the patient subsequently regains the capacity to understand the study procedures and provide informed consent, their consent must be obtained. Patients can be included in emergency situations if their condition precludes consent and no legal representative or close relative is available to provide consent. In this case, the investigator notes and justifies in the patient’s medical record that the patient was temporarily unable to receive the study information and provide informed consent, and that no legal representative or close relative could be reached. Written consent must subsequently be obtained from the patient, as soon as their clinical status allows.

#### Randomization

Randomization is performed during the first 48 hr of septic shock after the development of at least one criterion defining the failure grade of the RIFLE classification. Randomization takes place after verification of the eligibility criteria, following an online request by the investigator using Tenalea® software (Formsvision BV, Abcoude, Netherlands). This allocation is based on a minimization technique taking into account the center, age, sequential organ failure assessment (SOFA) score, site and type of infection. Patients are randomly assigned to one of the two groups in a 1:1 ratio. Due to the nature of the study intervention, blinding is not possible. However, the CIC-EC (Dijon) will manage all the data and generate blinded reports for the Data Safety and Monitoring Board. The investigators at the participating sites will remain unaware of study group outcomes until the database is locked.

### Study intervention

#### Initiation of RRT

Patients randomized to the early RRT arm will immediately undergo dialysis catheter placement. A maximum of 12 hr is allowed between the diagnosis of AKI and the actual initiation of RRT; this allows for the time required for catheter placement and installation of the RRT equipment, as well as any technical difficulties that may be encountered in clinical practice.

Patients randomized to the delayed RRT group will be closely monitored for 48 hr after randomization to identify as soon as possible the appearance of criteria requiring emergency RRT as defined above. If any of these criteria occur, dialysis catheters are placed and the RRT will be initiated as soon as possible.

In the absence of the appearance of criteria requiring emergency RRT, patients randomized to the delayed RRT group will undergo insertion of the dialysis catheter and initiation of RRT at least 48 hr after the diagnosis of AKI, with a maximum margin of 12 hr allowed (i.e. up to 60 hr maximum), justified by the same technical reasons mentioned above for the early RRT group.

If a patient in the delayed RRT group recovers normal renal function (defined according to the criteria for recovery of renal function listed above) within the 48 hr after randomization, the investigator is free to choose the most suitable treatment and is therefore not obliged to dialyze the patient, but must, in this case, document the absence of RRT and the normalization of renal function.

### Data collection

Detailed clinical procedure-related RRT parameters, and physiologic and laboratory data will be collected. Blood and urine will be collected at baseline, 12, 24, 36 and 48 hr and once daily thereafter until the study participants are discharged from ICU. Data will be collected on standardized case report forms (CRFs). All adverse events will be recorded on the CRF on specific pages reserved for this purpose. A simplified procedure will be used for foreseeable adverse events (hemodynamic instability, rhythm disturbances, electrolyte or acid-base imbalance, and bleeding events related to hemodialysis catheters or anticoagulants). Adverse events will be considered as serious if they cause death, are life-threatening, cause hospitalization (or prolongation of initial hospitalization), cause disability or cause permanent damage, a congenital anomaly or birth defect. Investigators must report any serious adverse event to the sponsor (the University Hospital of Dijon, France) promptly by email or telephone, followed by a written report within 48 hr. Completed CRFs will be returned to the CIC-EC and entered into a secured central database for independent quality control and centralized analysis.

Clinical data obtained will include demographic characteristics, comorbidities and prescribed and current drug therapy. Details of admission diagnoses, surgical status, and dates of hospital and ICU admission will be recorded. Detailed data on AKI and on septic shock will be recorded on the date of enrollment: interventions (i.e. mechanical ventilation, vasoactive drugs and fluid therapy), hemodynamics (i.e. blood pressure, heart rate and central venous pressure), nephrotoxic drugs (e.g. aminoglycosides, radiocontrast agents and hydroxy-ethyl starch), details of sepsis (i.e. site of infection and effectiveness of antimicrobial therapy) and acute physiology (i.e. components of severity of illness and SOFA score, simplified acute physiology score (SAPS II), urine output, fluid balance and secondary non-kidney organ dysfunction). During the trial, data will be collected on daily urine output, fluid balance, electrolytes, acid-base status, serum creatinine and urea, and components of the SOFA score. Data will be collected each day on whether the primary endpoint (mortality) has occurred, for evidence of any secondary endpoints and for criteria for trial discontinuation. Finally, any study protocol violations will be recorded.

All enrolled patients will be followed to determine mortality, continued need for RRT or renal recovery until death or discharge from hospital (whichever occurs first) and at 28, 90, 180 days and 1 year after randomization. Health-related quality of life will be assessed using the EQ-5D form by telephone contact at 90 days and 1 year. The EQ-5D is a generic health-related quality of life measurement instrument comprising five multiple choice questions, and a 100-point overall health state visual analogue scale (VAS) [30]. The questions concern mobility, self-care, daily activities, pain, anxiety and depression. The respondent is required to select one of three ordinal answers best describing their health state in relation to these five domains. Scores are then converted to an overall utility score, with 0 representing death, and 1 representing perfect health.

### Statistical analysis

All enrolled patients will be included in the main population analysis. The primary analysis will be by intention to treat (ITT). The characteristics of the two groups will be compared using the usual univariate tests (chi-squared or Fisher’s exact test for categorical variables, and analysis of variance or Mann–Whitney test for quantitative variables, as appropriate). The main comparison of the proportion of deaths at 90 days in both treatment groups will be performed using Fisher’s exact test, with secondary analysis by the Kaplan–Meier method, comparison using the log rank test, and Cox regression.

For the per-protocol analysis, patients will be grouped according to the time of initiation of the RRT. If RRT is initiated within 12 hr after the onset of the failure stage according to the RIFLE classification, then the patient will be included in the early RRT group. Conversely, if RRT is initiated >12 hr beyond this period, the patient will be included in the delayed RRT group. The per-protocol analysis will use the same techniques as the ITT analysis.

If the ITT and/or per-protocol analyses show a benefit of early (<12 hr) initiation of RRT, a secondary analysis will be performed using an extension of the proportional hazards model proposed by Abrahamowicz and MacKenzie [31] to estimate the non-proportional and non-log-linear effects, taking into account the exact time since initiation of RRT. This model will make it possible to trace the effect of initiation of RRT over time, thus visualizing whether early initiation results in lower risk of death after adjusting for major confounders, taking into account any non-proportional and/or non-log-linear effects. The main conclusion of the test will only cover the ITT analysis. Two interim analyses will be performed after the inclusion of, respectively, 200 and 400 patients.

Safety will be analyzed by assessing the frequency of arterial hypotension requiring the introduction or increase of noradrenaline, pulmonary edema due to overload, cardiac arrhythmias (ventricular tachycardia, ventricular fibrillation, torsade de pointes and atrio-ventricular block grade III), severe metabolic disorders and blood transfusions (≥3 units of packed red blood cells) in both groups and comparing rates using the appropriate tests, with an alpha risk set at 0.05.

The frequency of death within 90 days after randomization will be recorded in both groups and compared using a chi-squared test with alpha risk set at 0.0001 for both interim analyses, according to the method proposed by Peto [32] so as not to change the level of significance of the final primary analysis.

All analyses will be performed under the guidance of the Data Safety Monitoring Board, who will decide on the necessity to recommend premature interruption of the study to the Steering Committee, according to the results of the interim analyses.

#### Secondary analyses

1. An analysis identical to the primary analysis, studying mortality at 28 days taking an alpha risk of 0.05.

2. Average length of ICU stay and in-hospital stay will be described and compared for survivors.

3. The number of days without mechanical ventilation and the number of days without RRT in the two groups will be compared. Deceased patients are considered as having a number of days without mechanical ventilation and without RRT equal to zero. The numbers of days will be compared in both groups using a Mann–Whitney test.

4. Dependence on dialysis at hospital discharge will be compared between groups using the chi-squared or Fisher’s exact test. If the groups appear imbalanced in terms of the pre-existence of non-dialyzed chronic renal failure or administration of nephrotoxic drugs, then logistic regression will be performed to adjust for these factors.

5. The frequency of occurrence of at least one episode of hypotension requiring the introduction or increase of noradrenaline, pulmonary edema due to overload, cardiac arrhythmias (ventricular tachycardia, ventricular fibrillation, torsade de pointes and atrio-ventricular block grade III), severe metabolic disorders and blood transfusions (≥3 units of packed red blood cells) will be compared between groups using the chi-squared or Fisher’s exact test.

6. The number and characteristics of patients in the delayed RRT group for whom the occurrence of a criterion for emergency RRT was observed will be described.

These comparisons will be performed by ITT and per protocol. All analyses will be performed using SAS Version 9.2 (SAS Institute Inc, Cary, NC, USA) by the team of statisticians at the CIC-EC of the University Hospital of Dijon, France. The significance level is set at 0.05 for all final analyses.

### Data safety and monitoring

The trial has an independent data safety and monitoring committee with four members: two medical doctors (one intensive care specialist and one nephrologist), one statistician and one pharmacologist. These people are independent of the principal investigator and have no financial, scientific or other conflict of interest with the trial. Current or past collaborators of the principal investigator are not eligible to serve on the committee. Members of the committee have expertise in intensive care (acute kidney injury, septic shock and RRT), clinical trial methodology and biostatistics.

### Ethical considerations

This study was evaluated and received authorization from the Ethics Committee (Comité de Protection des Personnes (CPP) Est I) under the reference number 2012-A00519-34 and from the French National Health Products Safety Agency (Agence National de Sécurité des Médicaments et des Produits de Santé, ANSM). Data processing methods are reported to the national authority for the protection of privacy and personal data (Commission Nationale Informatique et Liberté, CNIL). Specific insurance for the study has been arranged with the Hospital Mutual Insurance Company, policy no 129234.

## Discussion

This protocol for a randomized controlled study investigating the impact of the timing of RRT initiation should provide an answer to a key question in the management of patients with AKI in the context of septic shock, for whom the mortality rate remains close to 60% despite improved understanding of the pathophysiology and recent therapeutic advances. For the last ten years, the key message in the treatment of septic shock has been the optimization of initial management. Taking a closer look at this concept, it seems legitimate to hypothesize that early treatment of AKI during septic shock could improve prognosis for patients.

It might have been preferable to initiate RRT at an even earlier stage of AKI; however, at the injury stage of the RIFLE classification, only 12% of patients have been shown to need RRT in observational studies [33], and it is not acceptable in ethical terms to initiate RRT so early. To the best of our knowledge, no novel biomarker or other parameter yet makes it possible to detect the need for RRT at an earlier stage, and with sufficient accuracy.

For the delayed group, we chose a time delay of 48 hr as we considered this to be sufficiently long to achieve hemodynamic stability in septic shock, and to observe a spontaneous improvement in renal function, in the best-case scenario. In case of progression to AKI, it is not ethically acceptable to delay RRT by any more than 48 hr. Lastly, we chose to use the term ‘delayed’ rather than ‘late’, because ‘late’ has previously been used to refer to much longer time spans, such as beyond 5 days in one study [19].

Clinical research on AKI has traditionally been difficult due to the lack of standardized definitions. A recent survey revealed the use of at least 35 definitions in the literature [34]. To minimize this problem and to generalize the results easily, we chose the RIFLE classification, which is widely known and has been validated by large series from Europe [35], the USA [36] and Australia [37], each including several thousand patients. The Acute Kidney Injury Network (AKIN) classification [38] did not substantially modify the definition of the failure stage (stage 3 in the AKIN classification) [39,40]. The recent classification proposed by the *Kidney Disease: Improving Global Outcomes* (KDIGO) guidelines had not been published at the start of our study [29].

The presence of criteria for emergency dialysis could be a barrier to good compliance in our study. To avoid this problem, we decided to exclude from the study patients who fulfilled emergency RRT criteria at ICU admission before randomization, although this could actually be a selection bias by limiting the inclusion of the most seriously ill patients. On the other hand, emergency criteria after randomization pose no problem for the study, because RRT will be initiated immediately regardless of the randomization group. The data will then be analyzed by ITT but also per protocol.

Another situation that may arise is that patients in the delayed RRT group recover adequate kidney function before RRT is initiated, and are not on RRT at 48 hr, but subsequently renal function gradually alters and RRT is started later. These patients will still be included in the ITT analysis and considered as adverse events, but a subgroup analysis of these patients is planned, since they constitute a particularly serious population with a very high mortality and therefore, merit evaluation.

### Potential challenges with the trial

If this study confirms the hypothesis that early RRT treatment is superior in the specific condition of AKI in septic shock patients, then this may help to reduce mortality for this very serious disease. The absence of a positive result will encourage us to focus our efforts on other aspects of the early management of these patients by delaying the initiation of renal supplementation.

## Trial status

Recruitment is ongoing (200 patients included as of 16 June 2014).

## Abbreviations

ADQI: Acute Dialysis Quality Initiative; AKI: acute kidney injury; AKIN: Acute Kidney Injury Network; CIC-EC: Centre for Clinical Investigation and Clinical Epidemiology of the University Hospital of Dijon, France; CRF: case report form; EQ-5D: European Quality of Life Five Dimensions questionnaire; ICU: intensive care unit; ITT: intention to treat; KDIGO: Kidney Disease: Improving Global Outcomes; MDRD: modification of diet in renal disease; RIFLE: risk, injury, failure, loss, end-stage kidney disease; RRT: renal replacement therapy; SAPS II: simplified acute physiology score; SLED: sustained low-efficiency dialysis; SOFA: sequential organ failure assessment; VAS: visual analogue scale.

## Competing interests

The authors declare that they have no competing interests.

## Authors’ contributions

SDB, CB and JPQ designed the trial, and obtained funding for the trial. SDB drafted the manuscript. SDB, CB, MM, RB and JPQ provided a critical revision of the manuscript. All authors read and approved the final manuscript.
